# Theoretical and Practical Aspects in the Use of Bretschneider Cardioplegia

**DOI:** 10.3390/jcdd9060178

**Published:** 2022-06-02

**Authors:** Claudiu Ghiragosian, Marius Harpa, Alexandra Stoica, Flămînd Oltean Sânziana, Radu Bălău, Hussam Al Hussein, Ghiragosian-Rusu Simina Elena, Radu Mircea Neagoe, Horațiu Suciu

**Affiliations:** 1 Department of Surgery IV, George Emil Palade University of Medicine, Pharmacy, Science, and Technology of Targu Mures, 540142 Targu Mures, Romania; claudiu.ghiragosian@umfst.ro (C.G.); alexandra.stoica92@yahoo.com (A.S.); flamand.sanzi@yahoo.com (F.O.S.); radu.balau@umfst.com (R.B.); horatiu.suciu@umfst.ro (H.S.); 2 Department of Anatomy-Embriolog M1, George Emil Palade University of Medicine, Pharmacy, Science, and Technology of Targu Mures, 540142 Targu Mures, Romania; hussam.al-hussein@umfst.ro; 3 Department of Pediatrics III, George Emil Palade University of Medicine, Pharmacy, Science, and Technology of Targu Mures, 540142 Targu Mures, Romania; simina.ghiragosian-rusu@umfst.ro; 4 Department of Surgery II, George Emil Palade University of Medicine, Pharmacy, Science, and Technology of Targu Mures, 540142 Targu Mures, Romania; neagoerm@gmail.com

**Keywords:** Custodiol, cardioplegia, cardiac arrest

## Abstract

The race for an ideal cardioplegic solution has remained enthusiastic since the beginning of the modern cardiac surgery era. The Bretschneider solution, belonging to the “intracellular cardioplegic” group, is safe and practical in myocardial protection during ischemic time. Over time, some particular concerns have arisen regarding the effects on cardiac metabolism and postoperative myocardial functioning. This paper reviews the most important standpoints in terms of theoretical and practical analyses.

## 1. Introduction

The introduction of the cardiopulmonary bypass (CBP) technique in 1951 by Gibons [1], aided by hypothermic maneuvers in 1950 by Bigelow [2], helped cardiac surgeons safely tackle pathologies. The bloodless surgical fields needed in the majority of cardiac interventions have been achieved using heart–lung machines. There are multiple variations regarding the extracorporeal circuit layouts, which are continuously evolving, and specific to certain pathologies or the surgeon’s choices.

Cardioplegic solutions are fundamental cardioprotective pharmacological agents used to attain two secondary conditions for heart surgery: electromechanical stillness and cardioprotection. As a result, surgical accuracy has improved and myocardial injury could be prevented. The term cardioplegia (*cardio* “heart” and *plegia* “paralysis”) was first introduced by Lam in 1957, suggesting the role of electromechanical quiescence [3]. The first step to a cardioplegic solution was taken in 1955 by Melrose [4], who observed: “elective reversible cardiac arrest” after infusion of highly concentrated potassium citrate. Over time, these solutions were perfected in terms of composition, dosage, temperature, and the adequate administration pathway; now, surgeons can tailor the optimal cardioprotective strategy for each pathology [5]. This continuous improvement has led to the omnipresence of cardioplegias in modern cardiac surgery, where a variety of cardioplegic formulas exist, each configuration with a specific action. There are many debates regarding the effectiveness of each solution [6,7,8,9].

Over the last decade, the average age of patients who were subject to cardiac intervention increased from 55.8 years to 68.8 years [10,11]. As a consequence, cardioprotective solutions that were developed for younger (and less ill) patients are now under significant stress. This is due to the advanced impairments of functional and structural aspects of the heart, as well as prolonged ischemic times [12]. For the abovementioned situations, our current cardioprotective regimes (in a universal implementation) are less optimal choices. Among the most important elements in favor of increased perioperative risks, there is age > 70, female sex, NYHA III/IV, ejection fraction <50%, pulmonary hypertension, chronic lung disease, extracardiac arteriopathy, renal impairment, and insulin-dependent diabetes [13]. From the cardioprotective point of view, left ventricular hypertrophy is one of the most deleterious changes in the cardiac structure, owing to the inadequacy in cardioplegic distributions in such cases.

It is complex to find a balance between doing as much as needed and as little as possible in cardiac surgery. Taking that into consideration, the administration of a single brief cold cardioplegia that offers up to 3 h of cardioprotection during cross-clamping aids in technicality, reducing the ischemic time during complex interventions [14,15,16]. This paper reviews the clinical principles of cardioprotection, solely focusing on histidine–tryptophan–ketoglutarate (HTK), branded as Custodiol^®^ by Essential Pharmaceuticals LLC [17]. After reviewing the latest articles, we noticed that some surgeons are reluctant to use Custodiol in complex cardiac interventions, especially CABG. There is uncertainty about one key advantage: myocardial protection during a long period with each infusion. There are concerns regarding the uniform distributions of cardioplegia in severe coronary stenosis, hemodilution, hyponatremia, myocardial edema, rhythm disorders, etc. It is necessary to highlight the clinical data behind these controversies when using the Bretschneider solution, in the interest of making full use of the specific advantages in the safest manner.

## 2. Principles of the Cardioprotection Strategies

Regardless of the preferred cardioplegic solution, all types of agents must fulfill certain conditions, as listed below:

1. Replenish the energetic deposits to meet the metabolic demands of the heart during aortic cross-clamping.
2. Rapid diastolic heart arrest.
3. Rheologic properties for proper dispersion to the entire myocardium.
4. Increased osmolarity to counteract myocardial edema secondary to ischemia.
5. Buffering effect to hinder secondary acidosis.
6. Reversibility.
7. No (or reduces) adverse effects.
8. Induction of hypothermic state, for specific cardioprotective solutions.

Based on these principles, several cardioplegic solutions were developed, analyzed, and improved in the last fifty years. These are divided into intracellular and extracellular cardioplegias. Intracellular agents are HTK, University of Wisconsin (UW), and Euro-Collins. UW, originally used in general surgery, has proven effective over time in intra-abdominal organ transplants and has gradually been used in heart transplants. It is a solution with a low concentration of Na^+^ (20 mmol/L), a high concentration of K^+^ (125 mmol/L), and increased viscosity compared to other crystalloid solutions. Although there were concerns about its uniform distribution in the myocardium and endothelial damage due to high K^+^ content, studies have suggested the reliability of this cardioplegia. The Euro-Collins solution, and its predecessor, the Collins solution (also a preserving agent for intra-abdominal organ transplantation), are intracellular cardioplegias used mainly for heart preservation during transport. Currently, few cardiac centers regularly use it for correction procedures. Extracellular cardioplegias are: St. Thomas no. 1 and no. 2, Buckberg, and del Nido. St. Thomas has been promoted at St. Thomas Hospital in London since 1976; it suffered some changes regarding electrolyte concentration. In 1981, an improved formula emerged—St. Thomas no. 2 (Plegisol, Abbott Laboratories, North Chicago). Del Nido cardioplegia was initially developed for pediatric interventions. It is a low-glucose hyperpolarizing crystalloid solution delivered in a combination of 1:4 with autologous blood. Although research on its use in adult cardiac surgery is scant, nowadays, in some centers, Del Nido cardioplegia is implemented successfully in adult cardiac surgeries. In the 1970s, autologous blood was used for the first time as a part of cardioplegic solutions, namely in Buckberg cardioplegia, to reduce the impact of the reperfusion phenomenon, containing potassium chloride as a depolarizing agent in a dextrose-based solution.

To understand the role of cardioprotective regimes, one has to consider that cardioplegia must protect the myocardium from adverse effects secondary to the ischemic event (anaerobic state) and from the subsequent reperfusion phenomenon.

Ischemia: a mismatch between the myocardial blood flow supply and myocardial oxygen demand, leading to myocyte impairment, which is initially reversible through adaptive response. If there is no perfusion for several minutes, an irreversible structural injury follows. Secondary to tissue acidosis, there is an accumulation of intracellular calcium and reactive oxygen species (ROS); these pathophysiological changes mainly affect microcirculation. This chance leads to ultrastructural alterations and cellular apoptosis [18,19].

The reperfusion event (hypoxia–reoxygenation phenomenon): in this case a growing body of evidence unveils microcirculation impairing with increased vascular permeability, myocytes swelling, edema, and inflammation with ventricular compliance reduction. These complex histopathological alterations are consequences of functional and structural endothelial damage. The coronary system is exposed to ROS, inflammatory response, and hyperkalemic agents [20,21]. There is a direct imbalance between endothelium-derived constricting factors: endothelin-1 (ET-1) and thromboxane A2 (TXA2), and relaxing elements, such as nitric oxide (NO) or endothelial-derived hyperpolarizing factor (EDHF), disturbing the synergistically-acting mechanisms between them [22]. NO, interfering in the modulation of the inflammatory response, displays an important role in enhancing coronary circulation, preventing platelet aggregation, and leukocyte adhesion secondary to vascular injury.

The ischemia–reperfusion (I–R) phenomenon is a double-staged event in which the initial hypoxic event can be roughly exceeded in terms of damage by the subsequent reperfusion [23]. The myocardial muscle is particularly susceptible to I–R in case of a long-lasting mitral insufficiency when there is increased activity of the xanthine oxidase enzyme, which causes mitochondrial oxidative stress [24,25]. As a central element in the mechanism of I–R, the coronary vascular network releases in the bloodstream endothelial cells, such as soluble thrombomodulin and the von Willebrand factor. Kuhn et al. quantified these circulating elements, noting that, secondary to warm cardioplegia infusion, there is a greater amount of components released as compared to cold agent administration, indicating greater vascular endothelial suffering [26]. As suggested, the cardioplegic temperature exhibits a certain influence on the coronary vascular network, but no study to date has been able to discern the extent of the impact on postoperative outcomes—it is still a matter of debate. Theoretical premises advocate warm and cold cardioplegia alike. The benefits of slowing cardiac metabolism induced by hypothermia (reducing oxygen consumption, maintaining the stock of high-energy phosphate) are well-known. This effect is maximized when cold cardioplegia is used. However, it is a bittersweet effect because low-temperature solutions interfere in the process of oxygen–hemoglobin dissociation, resulting in a leftward curve displacement in the oxygen–hemoglobin dissociation graphic. As a consequence, although hemoglobin has a higher affinity for oxygen, it unloads it more reluctantly. Some studies have shown that (to a certain degree) an acidotic environment during cross-clamping counteracts the effects of hypothermia in the oxygen hemoglobin dissociation process, shifting the curve to the right [27]. Additionally to systemic cooling, after cold cardioplegia infusion, the basal myocardial metabolic rate is reduced by a further 5–20%; by default, O₂ consumption is also diminished. As a generally accepted idea, low O₂ squandering means a higher content of creatine phosphate, aiding in contractile function recovery in the early postoperative period [28]. The meta-analysis by Fan et al. describes a similarity concerning the length of hospitalization following cardiac surgery and the incidences of adverse events between warm and cold cardioplegic groups, albeit the level of troponin I and CK-MB were significantly lower in the first group. Thereby, it can be deduced that (conceptually) there are no relevant hemodynamic or structural consequences that could have been suggested by the increased levels of cardiac enzymes [29,30]. Other studies emphasize superior results in warm group solutions for a particular cardiac intervention: early CABG after myocardial infarction, considering 30-day mortality and IABP employment [31]. While cardioplegic solutions are (for the most part) standard in composition, there are some distinctions regarding administration techniques that transcend both cardiac institutions as well as surgeons from the same cardiac center [32]. Further distinctions must be taken into account regarding operative techniques and skills and the lack of standardization across centers toward cardioprotection regimes—more than the natural disparities between patients (i.e., comorbidities, biological age, ethnicity). Thus, different results from certain debates emerge even with thorough randomization due to a multitude of confounders [33].

### Cardioplegic Administration

Cardioplegia is delivered from a container or with the help of a roller pump—part of the heart–lung machine. In the first case, we have a premixed solution in a container that can be pressurized or the solution can be infused at hydrostatic pressure, adjusting the height of the container over the heart to maintain a relative specific pressure. In the second scenario (infusion from the CBP), the machine offers some benefits, such as controlling the delivery pressure, flow rate, and temperature. Concerning the route for cardioplegic administration—there is the antegrade infusion (in the aortic root/coronary ostia, grafts), retrograde (coronary sinus), or combined. The total dose can be calculated in mL/kg, in minutes for the total infusion time, or, in some centers continuous administration is preferred. In the case of a competent aortic valve, infusion in the aortic root can be safely achieved. Cardioplegia is delivered via a venting catheter placed between the aortic clamp and the coronary ostia. Caution must be taken during cardioplegic administration concerning: pressure delivery, distension of the left ventricle (LV), palpatory assessment of the pressure in the ascending aorta, cessation of the electromechanical activity, and a solution washout through the LV venting catheter. High pressure of infusion can cause myocardial edema and coronary lesions; dilatation of the LV might induce postoperative LV disfunction.

Delivering the plegic solution directly into the coronary ostia is preferable in aortic insufficiency cases or if an intervention to the ascending aorta is planned. Therefore, there is a direct view to properly inspect the administration of the cardioplegic agent. As a shortcoming, after manipulation of calcified coronary ostia with the catheter tip, coronary embolization of debris or later ostial stenosis can occur. There are some distinct situations when direct canulation of the coronary ostia can be demanding:

- In 50% of the population, the conus arteries have separate small ostia near the right coronary ostia;
- Some patients with bicuspid valves have shorter left main coronary arteries; the possibility of malperfusion in the circumflex artery or the descending artery is high.

Perfusing through the free end of the saphenous—or the free arterial graft—after the distal anastomosis is complete provides a more homogeneous distribution in patients with severe coronary stenosis/occlusions.

Retrograde cardioplegia can be infused with a pressure of 30–50 mmHg using a smooth cuff, wire-wound body with a 10 to 15 Fr lumen inserted into the coronary sinus via a right arteriotomy or percutaneously inserted through the right internal jugular vein. This method of protecting the heart is beneficial in the hypertrophied LV, coronary atherosclerosis with severe stenosis/occlusions, or CABG reinterventions [34].

## 3. Bretschneider

Cardioprotective regimes are continuously evolving, given that the patient collectives requiring cardiac surgeries are becoming more demanding. The segment of high-risk patients with multiple cardiac lesions and comorbidities is shaped as life expectancy increases, with longer cardiopulmonary bypass times (CPTs), pushing the limits of currently available cardioprotection regimes. The current literature on cardioprotection mostly concludes the following: “further inquiry needed”. Thereby, certain hypotheses have arisen, based on which current cardioprotective protocols are less than ideal, especially concerning particular clinical layouts. There are less than ideal comprehensions of the intricate mechanisms in cardioplegia.

HTK, developed by the German physiologist Hans Jürgen Bretschneider at the University of Göttingen in the early 1970s (branded as Custodiol^®^ by Essential Pharmaceuticals, Durham, NC, USA, has been proposed as a substitute for blood agents and other conventional cardioplegias, with up to 3 h of myocardial protection in conjunction with hypothermia [35,36,37]. Surgeons can tackle laborious interventions (they gain time by reducing the total time of administering cardioplegia) [38,39,40,41,42]. For a significant period of time, this type of cardioplegia was forgotten. This was caused by the difficulty in understanding the complex biochemical mechanisms involved in inducing cardiac arrest, as well as the indifference by the cardiac surgery community concerning the time toward cardioplegia in general. Since the 1980s, the Bretschneider solution has been used in cardiac transplantation, due to its long-lasting cardioprotection properties. Current studies confirm the benefits of using Custodiol for allograft preservation. Regarding hemodynamic parameters, cardiac enzyme release, and 30-day mortality, Bretschneider cardioplegia provides similar results compared to cold blood cardioplegia, with built-in advantages (in terms of technicality and shortening ischemic times) [43].

HTK, a long-lasting single dose cardioprotective agent, improves the early results following cardiac surgery, involving cardiac arrest, especially in complex interventions with prologue CBP. As such, those highly invasive interventions are transformed into routines. Researchers, reviewing the efficiency of this cardioprotective agent, quantified it in the perioperative period of muscular deterioration (newly onset muscular hypokinesia, akinesia, or dyskinesia plus myocardial enzymes). In addition, ECG traces were analyzed for possible rhythm disorders or new Q-waves [44,45,46,47,48,49]. The term “micro-necrosis” refers to elevated values of cardiac enzymes in the absence of conventional markers for myocardial infarction (ECG, echocardiographic), occasionally an identifying mark for improper intraoperative cardioprotection. Resorting to echocardiography, one can inspect the interventricular septum; it is known as being the best imagistic marker for quantifying myocardial disfunction. It makes up 35–40% of the biventricular muscular substance and a half of the left ventricle. It is accountable for 80% of the right ventricle functions as a result of twisting and shortening movements [50]. As a consequence, an impaired septal function will develop right ventricular dysfunction, highlighted by measuring the tricuspid annular plane systolic excursion (TAPSE) and tricuspid annular plane systolic excursion velocity (‘S’) [51].

**Figure 1:**
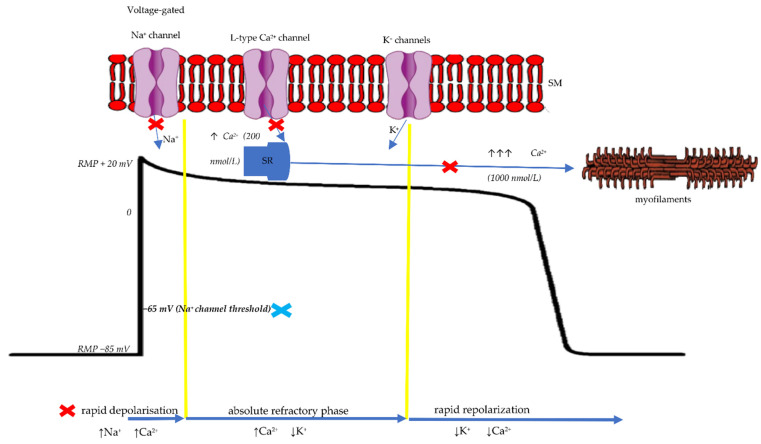
The action mechanisms of Bretschneider and St. Thomas cardioplegias. SR: sarcoplasmic reticulum; SM: sarcolemmal membrane; RMP: resting membrane potential; When impuls reaches the myocardial cell, the voltage-gated Na^+^ channels opens to permit Na^+^ ions to rapidly enter the cardiomyocyte via the electromechanical gradient (fast depolarization phase). The activation of Na^+^ channels is produced at an RMP of −65 mV. Further activation of L-type Ca^2+^ channels ocurrs (when RMP reaches −30 mV) with a slow infusion of Ca^2+^ cations (up to 200 nmol/L) that in turn, signals a larger amount of Ca^+^ to be released from the sarcoplasmic reticulum (calcium induced calcium release, up to 1000 nmol/L). This resulted cytosolic Ca^+^ cause the forming of electromechanical coupling (corresponding to the absolute refractory phase). Blue Cross Symbol: St. Thomas action-providing a large quantity of K^+^ cations, the membrane RMP stays “more positive” (−55 mV) as against to Na^+^ channel threshold of −65 mV (keeping it inactive). Red Cross Symbol: Custodiol action-hypocalcemia, induced by infusing a high amount of cardioplegia with minimal Ca^2+^ content, inhibits the L-type Ca^2+^ channels. As such, the formation of electromechanical coupling is abolished. The hyperpolarization effect extracellular hyponatremia prevents Na^+^ from opening blocking the rapid depolarizing phase, keeping the RMP at a level close to the value of resting potential. The Mg^2+^ content in both types of solutions acts as an L-type Ca^2+^ channel blocker, through Ca^2+^ dislocation [38,45].

The histidine–tryptophan–ketoglutarate cardioplegic solution is part of the so-called “intracellular” cardioplegic group; this collective label suggests a similarity between Na^+^ concentration in the Custodiol solution as in the myocardial cytoplasm. It has the purpose of arresting the heart in diastole acting through Na^+^ depletion of the extracellular space, resulting in hyperpolarization of the membrane, inhibiting the fast depolarization phase. In other words, HTK deprives the cardiomyocytes of the Na^+^ ions; as a result, there are no exchanges across the ion channels with no action potential formation. This mechanism of producing cardiac arrest distinguishes it from the process involved when using “extracellular” cardioplegias with a high amount of potassium chloride, inducing cardiac arrest by membrane depolarization (Figure 1).

The state of hyperpolarization represents the native condition of the cardiac cell when found in an inactive condition. Beginning with this evidence, in animal heart studies, hyperpolarization arrest has been obtained (acting on ATP-sensitive K^+^ channels). Subsequently, cardiac functioning was assessed after resuming cardiac activity compared to hearts arrested with depolarizing agents. The superiority of hyperpolarization solutions stands out, suggesting better protection when it comes to adequately arresting the heart [45,52].

Bretschneider, describing the benefits of every component in his newly devised cardioplegia, mentioned lower concentrations of prime electrolytes (Na^+^, K^+^, and Ca^2+^) compared with other cardioplegias [53]. By comparison, the hyperkalemic solution interferes in the production of the endothelium-derived relaxing factor, alters myocardial perfusion, and induces smooth muscle proliferation [54,55]. Moreover, in a study conducted by Ilker M. et al., the authors analyzed endothelial injury secondary to Bretschneider infusion versus cold blood cardioplegia in 50 CABG interventions. Postoperative low levels of endothelin-1 in the crystalloid group compared to the blood cardioplegic group demonstrated lower endothelial injuries [56].

Likewise, it was reported that there was an association between secondary systemic vasodilatation (due to hyperkalemic agents in the course of a cardiopulmonary bypass) and vasopressor agents, with subsequent adverse effects [57]. The Bretschneider cardioplegia, as an additional solute, contains histidine, which contributes as a buffering agent, improving cellular integrity during anaerobic glycolysis and performing as a free radical scavenger. Mannitol (an osmotic diuretic) was added to the HTK composition along with ketoglutarate, an intermediary in the Krebs cycle, to assist in ATP generation during reperfusion. Tryptophan acts as a membrane stabilizer. With a high concentration of scavengers in addition to the absence of leukocytes (in contrast to blood cardioplegia), injuries secondary to the I–R phenomenon are (in theory) somewhat diminished [58,59,60,61]. Table 1 presents the main differences among the most used cardioplegic solutions.

Inducing cardiac arrest usually implies slow infusion of the HTK solution at a temperature of 5–8 °C into the coronary system, with a hydrostatic pressure of 100 mmHg, which corresponds to approximately 140 cm above the level of the heart, depending on the perfusion kit set up. After cardiac arrest commences, the pressure of perfusion should be reduced to 40–50 mmHg, higher in cases presenting severe coronary stenosis. This reduction of pressure can be achieved by lowering the solution container to approximately 50 cm above the level of the heart. The Bretschneider solution can also be infused via a roller pump—this is lucrative in minimally invasive procedures due to the limited access [62,63,64,65,66]. Because the processes involved in membrane hyperpolarization are time- and volume-dependent, to ensure homogeneous equilibration, the total infusion time must be between 6 and 8 min. As a premise, a crystalloid-based cardioplegia has better rheology during infusion than its blood-based counterparts, resulting in a homogenous dispersion throughout the myocardial muscle, reduced shear stress, and vascular resistance.

Concerns have been raised regarding the subsequent hyponatremia, on account of a large quantity of low Na^+^ infusions [67,68]. With a concentration of 15 mmol/L of NaCl, HTK is, in effect, a hyponatremic compound and a rather hyperosmolar solution, given the fact that it has 310 mOsmol/L due to the abundance of amino acids. In 2012, Lindner et al. determined the impact of Custodiol administration on electrolyte homeostasis in 25 patients during minimally invasive aortic valve replacements. The mean decrease in plasma Na^+^ was 15 mmol/L as compared to the preoperative state, with a constant osmolality. Slight isotonic hyponatremia is considered a harmless condition and it should therefore not be treated aggressively, or not at all, since (managed excessively) it can lead to acute hypernatremia with deleterious repercussions [69,70].

The Bretschneider cardioplegia—preventing the exhaustion of energy constituents and preserving ultrastructural integrity—is suitable for cold static preservation of the heart at a temperature of 2–4 °C when transporting it to the recipient’s site, approved by the U.S. Food and Drug Administration [71,72,73,74]. Harnessing the pivotal role of the high buffering capabilities of HTK, tissue acidosis secondary to long-lasting ischemia decreases, as Kallerhof et al. observed when studying rat hearts following cold storage in an HTK solution. A close to normal pH improves the ATP concentration with better recovery of contractile force after aorta declamping. We have to consider that cellular integrity during ischemia rests on a considerable amount of energy [75]. Likewise, in the same experiment, it was noticed that improved myocardial relaxation after reperfusion was secondary to acetylcholine administration. This observation indicates that Custodiol exhibits a prevention effect against I–R side effects and a protective role in cardiac microcirculation integrity [76].

In our center, HTK is a ubiquitous cardioplegic agent, used in pediatric and adult cardiac interventions. It is generally avoided in patients with severe multi-vessel diseases and left ventricular hypertrophies; in these cases, heterogeneous distribution in the cardiac muscle is possible due to the low pressure during infusion. Recalling the principle addressed above, the effectiveness of this cardioplegia resides from homogeneous distribution during a certain amount of time. In the hypertrophic ventricle, it has been emphasized postmortem and in an in-vivo analysis of coronary microvascular dysfunction. These pathological changes consist of: thickening of the intimal and media layers, loss of the subendocardial coronary reserve with decreased capillary density, and an increased oxygen diffusion distance. As an auxiliary element, in these particular cases, the sub-optimal distribution of cold cardioplegia can be determined using intraoperative thermographic imaging. Cardioplegia administration—detecting warmer areas on the surface of the heart—can be promptly manipulated to prevent postoperative myocardial dysfunction [77].

In some early studies, the Bretschneider solution was considered the culprit for causing myocardial edema, especially in complex cardiac interventions with prolonged cardiac arrest and repeat administration [78,79]. The resulting myocardial edema could impair ventricular functioning due to wall stiffening, decreased diastolic compliance, interstitial tissue fibrosis, and could make the heart more susceptible to conduction disturbances or arrhythmias [80]. More recent studies advocate protection against myocardial edema with the use of HTK, mainly due to mannitol content [81]. However, this is not a comprehensively studied topic in the literature, likely due to dissimilarities in the practice patterns when managing fluid and osmolarity balance. More research must be conducted to verify these findings.

Certain concerns have been raised regarding the postoperative elevations of cardiac enzymes after using HTK (although no connections could be made between this observation and specific postoperative adverse events) [82]. Knowing the interactive relationship between the ischemic time and myocardial enzyme release, generally, in surgical interventions conducted under Custodiol protection—there was a trend for longer cardiac ischemia due to higher surgical complexity. Certainly, those laborious cases represent a desired destination for the use of HTK, showing a clear efficiency by reducing the discontinuance during CBT for cardioplegic infusion [83,84]. A second possible cause for the cardiac enzyme increase could be repeated defibrillation after aortic declamping [85]. In our experience, and corroborated with reports from other cardiac centers, when using Custodiol, the most frequent arrhythmias that occurred after resuming cardiac activity were ventricular fibrillation and ventricular tachycardia. Usually, they were prone to self-restraint, i.e., spontaneous defibrillation [86,87,88]. Sixty-five patients with reduced ejection fractions (<45%) undergoing mitral valve replacements were included in a survey at Ain Shams University Hospitals. In group A, the Bretschneider solution was used; in group B—cold blood cardioplegia. The incidence of ventricular arrhythmia after aortic declamping (with secondary defibrillation) was significantly increased in the first group. In group A, the mean ejection fraction after the valvular replacement was higher compared to group B (53.17 ± 7.73 compared with 49.06 ± 7.170) [89]. Other recent surveys did not find statistical differences in myocardial damage (Troponin-I, CK-MB) comparing the cardioplegic solution in question to others that were renowned [90,91,92,93].

Hemodilution is a distinct adverse effect iterated after the use of HTK [94,95]. Iatrogenic-induced hemodilution is prone to blood transfusions, electrolyte disturbances, and cardiac arrhythmias. Correctly managing the fluid balance is critical in cardiac surgery, especially in patients with cardiomyopathy and associated vascular or pulmonary disorders [96]. Our institution uses an additional hemofilter in every cardiopulmonary setup, regardless of the chosen type of cardioplegia. Apart from the cytokine purge, it allows us to control the fluid balance according to prominent factors (cardioplegia, fluid resuscitation, intravenous drugs) in conjunction with urinary flow, gender, and age.

## 4. Conclusions

Bretschneider’s cardioplegic solution is easy to use in any setup of the cardioprotective regime, especially in complex cardiac interventions. The constitutive elements are indispensable for achieving the supreme goal: ideal protection. There is no consensual conclusion yet as to whether they have succeeded in fulfilling this purpose. The inequalities between studies (concerning the collections of patients and the surgical techniques) have caused long-standing controversies.

There are other cardioplegic solutions available; suitable matching with a given pathology is placed solely on the surgeon. It is safe to say that HTK, similar to other agents, passes the test of time with certain advantages. However, it is prudent to state that the ideal cardioplegic solution is unknown.

## Figures and Tables

**Table 1:** Differences between the most used cardioplegic solutions.

*Cardioplegia*	*Bretschneider*	*Modified St. Thomas (Plegisol)*	*Del Nido*	*Blood Cardioplegia*
*Type*	Intracellular	Extracellular	Extracellular	Extracellular
*Acting mechanism*	Hyperpolarization	Depolarization	Hyperpolarization	Depolarization
*Delivery*	Antegrade/Retrograde	Intermittent; Antegrade/ Retrograde/ Combined	Antegrade/Retrograde	Continuous/Intermittent; Antegrade/Retrograde/ Combined
*Cardioprotective time per administration*	0–120′ (Up to 240′ for Allograft Preservation)	20–30′	90′	20′
*Composition* *(mmol/L Unless Otherwise Indicated)*	Crystalloid Na^+^ 15 K^+^ 9 Mg^2+^ 4 Ca^2+^ 0.015 Mannitol 30 Histidine 198 Tryptophan 2 Ketoglutarate 1	1:4 Blood: Crystalloid Na^+^ 110 K^+^ 16 Mg^2+^ 16 Ca^2+^ 1.2 NaHCO_3_ 10	1:4 Blood: Crystalloid 1 L Plasma-Lyte+ Na^+^ 140 K^+^ 26 Mg^2+^ 3 Cl^−^ 98 Lidocaine 130 mg	4:1 or 2:1 Blood: Crystalloid Using 4:1 Ratio + Addition K^+^ 80 mEq Na^+^ 30 mEq 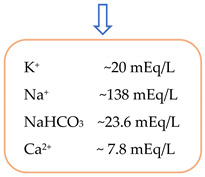
*Temperature*	5–8 °C (2–4 °C for Allograft Storage)	4–8 °C	8–12 °C	Cold (Buckberg) Tepid/Warm (Calafiore)
*Dosage*	1 mL/min./Gram-Estimated-Heart-Weight (6 to 8′)	Induction: 12 mL/kg (Total Body Weight) Maintenance: 10 mL/kg	20–30 mL/kg Total Body Weight	Induction: 10–12 mL/kg Total Body Weight Maintenance: 6–8 mL/kg

Note: These values can vary slightly, depending on the cardiac center or surgeon. The electrolyte content in the Del Nido solution is comparable with the one found in the extracellular space. There are two St. Thomas solutions: 1 (MacCarthy) and 2 (Plegisol). Both produce rapid cardiac arrest thanks to high concentrations of K^+^. With low concentrations of Na^+^ and Ca^2+^ ions, Custodiol cardioplegia inhibits action potential formation, producing diastolic cardiac arrest. The ‘’blood cardioplegia’’ group includes solutions such as Calafiore or Buckberg, both with “microplegia” alternatives.

## Data Availability

Not applicable.
